# Associations between Metabolomic Biomarkers of Avocado Intake and Glycemia in the Multi-Ethnic Study of Atherosclerosis

**DOI:** 10.1016/j.tjnut.2023.07.013

**Published:** 2023-08-09

**Authors:** Alexis C. Wood, Mark O. Goodarzi, Mackenzie K. Senn, Meghana D. Gadgil, Goncalo Graca, Matthew A. Allison, Ioanna Tzoulaki, Michael Y. Mi, Philip Greenland, Timothy Ebbels, Paul Elliott, Russell P. Tracy, David M. Herrington, Jerome I. Rotter

**Affiliations:** 1 USDA/ARS Children’s Nutrition Research Center, Baylor College of Medicine, Houston, TX, United States; 2 Division of Endocrinology, Diabetes, and Metabolism, Department of Medicine, Cedars-Sinai Medical Center, Los Angeles, CA, United States; 3 The University of Texas Health Science Center at Houston School of Public Health, Houston, TX, United States; 4 Division of General Internal Medicine, Department of Medicine, University of California, San Francisco, CA, United States; 5 Section of Bioinformatics, Division of Systems Medicine, Department of Metabolism, Digestion and Reproduction, Faculty of Medicine, Imperial College London, London, United Kingdom; 6 Division of Preventive Medicine, Department of Family Medicine, University of California, San Diego, La Jolla, CA, United States; 7 Department of Hygiene and Epidemiology, University of Ioannina Medical School, Ioannina, Greece; 8 Department of Epidemiology and Biostatistics, Imperial College London School of Public Health, London, United Kingdom; 9 Division of Cardiovascular Medicine, Beth Israel Deaconess Medical Center, Boston, MA, United States; 10 Cardiovascular Institute, Beth Israel Deaconess Medical Center, Boston, MA, United States; 11 Departments of Preventive Medicine and Medicine, Feinberg School of Medicine, Northwestern University, Chicago, IL, United States; 12 Biomolecular Medicine, Section of Bioinformatics, Division of Systems Medicine, Department of Metabolism, Digestion and Reproduction, Imperial College London, London, United Kingdom; 13 Laboratory for Clinical Biochemistry Research, University of Vermont, Burlington, VT, United States; 14 Section on Cardiovascular Medicine, Department of Internal Medicine, Wake Forest School of Medicine, Medical Center Boulevard, Winston-Salem, NC, United States; 15 Department of Pediatrics, The Institute for Translational Genomics and Population Sciences, The Lundquist Institute for Biomedical Innovation at Harbor-University of California, Los Angeles Medical Center, Torrance, CA, United States

**Keywords:** biomarker, type 2 diabetes, dysglycemia, metabolomics, personalized nutrition, MUFA, avocado

## Abstract

**Background:**

Avocado consumption is linked to better glucose homeostasis, but small associations suggest potential population heterogeneity. Metabolomic data capture the effects of food intake after digestion and metabolism, thus accounting for individual differences in these processes.

**Objectives:**

To identify metabolomic biomarkers of avocado intake and to examine their associations with glycemia.

**Methods:**

Baseline data from 6224 multi-ethnic older adults (62% female) included self-reported avocado intake, fasting glucose and insulin, and untargeted plasma proton nuclear magnetic resonance metabolomic features (metabolomic data were available for a randomly selected subset; *N* = 3438). Subsequently, incident type 2 diabetes (T2D) was assessed over an ∼18 y follow-up period. A metabolome-wide association study of avocado consumption status (consumer compared with nonconsumer) was conducted, and the relationship of these features with glycemia via cross-sectional associations with fasting insulin and glucose and longitudinal associations with incident T2D was examined.

**Results:**

Three highly-correlated spectral features were associated with avocado intake at metabolome-wide significance levels (*P* < 5.3 ∗ 10^–7^) and combined into a single biomarker. We did not find evidence that these features were additionally associated with overall dietary quality, nor with any of 47 other food groups (all *P* > 0.001), supporting their suitability as a biomarker of avocado intake. Avocado intake showed a modest association only with lower fasting insulin (*β* = –0.07 ^+/-^ 0.03, *P* = 0.03), an association that was attenuated to nonsignificance when additionally controlling for body mass index (kg/m^2^). However, our biomarker of avocado intake was strongly associated with lower fasting glucose (*β* = –0.22 ^+/-^ 0.02, *P* < 2.0 ∗ 10^–16^), lower fasting insulin (*β* = –0.17 ^+/-^ 0.02, *P* < 2.0 ∗ 10^–16^), and a lower incidence of T2D (hazard ratio: 0.68; 0.63–074, *P* < 2.0 ∗ 10^–16^), even when adjusting for BMI.

**Conclusions:**

Highly significant associations between glycemia and avocado-related metabolomic features, which serve as biomarkers of the physiological impact of dietary intake after digestion and absorption, compared to modest relationships between glycemia and avocado consumption, highlights the importance of considering individual differences in metabolism when considering diet-health relationships.

## Introduction

Diets to support optimal glucose homeostasis should be rich in whole grains, high in fiber, low in saturated fats (<7% of calories), and contain moderate amounts of MUFA [1]. As half an avocado contains almost 5 g of fiber and 6.7 g of MUFA [2], this fruit offers promise for supporting glucose homeostasis and contributing to a diet that reduces risk of type 2 diabetes (T2D).

We have previously reported, in large-scale epidemiological studies, that avocado intake is associated with lower glucose concentrations over an approximately three month period [assessed by hemoglobin A1c (HbA1c)], lower fasting glucose, and improved insulin homeostasis following an oral glucose challenge [3], as well as lower rates of incident T2D [4], similar to results found in avocado feeding studies [5,6]. In addition, prior avocado-glycemia associations were sensitive to individuals’ metabolic health, as they differed between individuals with normoglycemia compared with pre-diabetes compared with T2D [3,4]. This suggests an approach that is sensitive to individual responses to dietary intake (“personalized nutrition”) is needed to fully understand the role of avocado intake in dysglycemia.

Metabolomic data can serve as a key tool in such investigations. The plasma metabolome reflects, in part, how dietary intake is digested, processed, and absorbed (i.e., metabolized). We have theorized that this ability to capture individual responses to diet is one of the reasons why, in our prior investigation, diet-related metabolites (in this case, metabolites associated with unprocessed red meat intake, [7]) showed associations with health outcomes (markers of inflammation) that were orders of magnitude larger than the associations between dietary intake itself and the same outcomes [7].

Thus, to better understand associations between avocado intake and T2D risk, the current analyses sought to identify metabolomic features associated with habitual avocado intake and examine their associations with contemporaneous glycemia (fasting glucose and insulin) and incident T2D. We hypothesized that metabolites associated with avocado intake would be associated with lower fasting glucose and insulin and lower rates of incident T2D and that the relationships of avocado-related metabolomic features to glycemia and incident T2D would be stronger in those with dysglycemia.

## Methods

### Study population

**FIGURE 1:**
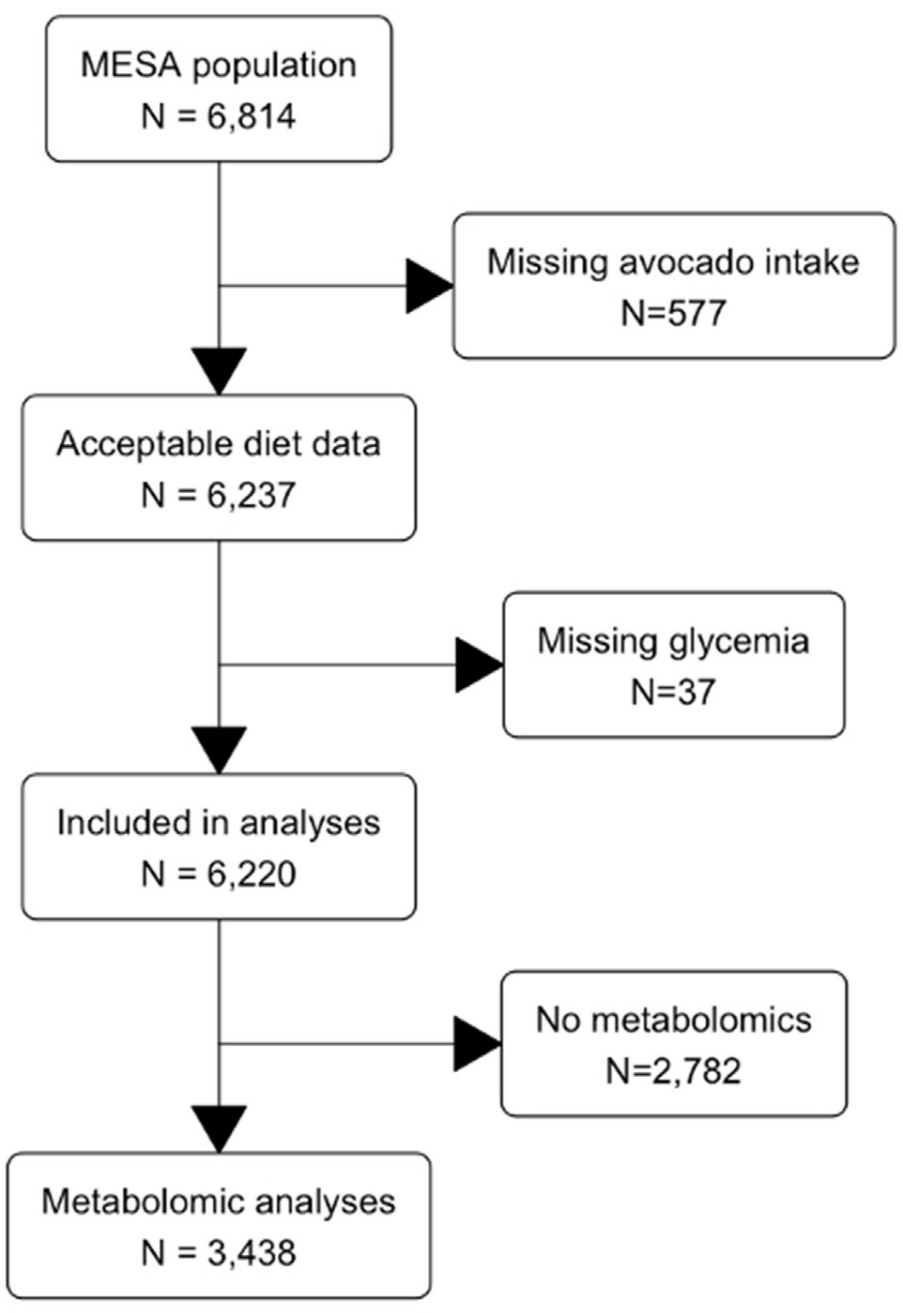
Participant flow diagram. MESA, multi-ethnic study of atherosclerosis.

The current analyses used data from the Multi-Ethnic Study of Atherosclerosis (MESA). Recruitment methods and study procedures for MESA have been previously described [8]. In brief, MESA consists of 6814 men and women, aged 45–84 y, who were free of clinical CVD at baseline (2000–2002), and self-reported their ancestry as White (*N* = 2623), black (*N* = 1891), Hispanic (*N* = 1496), or Asian (*N* = 804) (Figure 1). Participants were recruited from 6 sites across the United States (Baltimore County, MD; Chicago, IL; Forsyth County, NC; New York, NY; Los Angeles County, CA; and St. Paul, MN) for a baseline examination conducted between 2000 and 2002. Participants have been followed up at ∼ 18-mo intervals, with the most recent follow-up data on T2D incidence available from examination 6, which was conducted between 2016 and 2018. Participants without avocado intake data (*N* = 577, Figure 1) and/or baseline dysglycemia data (an additional *N* = 37; Figure 1) were excluded, leaving a final sample for analysis of N = 6220 (Figure 1). Metabolomics data were available for a randomly selected subsample of 3438 participants (Figure 1).

### Measures

#### Dietary intake

Usual dietary intake over the past year was assessed at the baseline clinic examination using an FFQ. The MESA FFQ is a modified version of the Insulin Resistance Atherosclerosis Family Study FFQ, which was validated in non-Hispanic White, Hispanic, and African American United States populations [9,10]. The Insulin Resistance Atherosclerosis Family Study FFQ was modified better to capture the intake of Chinese-American populations for MESA, and within MESA, has shown criterion and predictive validity [11,12]. The MESA FFQ asked about intake frequency and average serving size for 120 foods (including mixed dishes such as chow mein). Nine frequency options were given that ranged from “rare or never” to a maximum of “≥2 times/d” for foods and a maximum of “≥6 times/d” for beverages. Food and beverage questions from the FFQ were categorized into 47 food groups (including avocado) based on similar classifications (e.g., fruit compared with vegetables) and nutrient characteristics (for details on the weighted contribution of individual foods to each food group, see Supplemental Table 1).

#### Avocado intake

Avocado intake is an infrequently consumed food in the United States population [13]. Because of the zero inflation in the distribution, avocado intake was dichotomized into nonconsumers (no intake) and consumers (some reported intake) prior to analysis.

#### Diet quality

In analyses that attempted to understand the extent that any associations between spectral features and avocado intake were attributable to an overall healthier diet, a diet score reflecting adherence to a Mediterranean-style diet was added as a covariate to association models using a previously published scoring system [[14], [15], [16]]. To calculate this score, reported intakes for the following 9 components were cut at the sex-specific median and scored 0 or 1: *1*) vegetables, *2*), legumes *3)* fruit, *4)* nuts*, 5)* whole grains and *6*) fish which were positively scored, and *7*) red meat, *8)* dairy, and *9)* saturated to unsaturated fat ratio, which was negatively scored. Scores for these components were summed, and a score of 1 added for men who reported 1–2 alcoholic drinks/d (3.5–17.5 g of ethanol) on average, and for women who reported ∼1 alcohol drink/d (3.5–10.5 g of ethanol), such that the final score had a range of 0–10.

#### Proton NMR untargeted metabolomics

Using stored fasting serum samples from the baseline assessment, untargeted metabolomic profiles were generated for 3955 randomly selected participants via a standard proton NMR (1H NMR) 1D NMR spectrum with water suppression and a T2-edited spectrum that used a Carr-Purcell-Meiboom-Gill sequence. Serum samples were at –80°C until analysis. After thawing, 300 *μ*L of serum was mixed with 300 *μ*L of phosphate buffer in Eppendorf tubes (Eppendorf, AG, Germany), and subjected to centrifugation, then kept at 4°C until analysis. For each 96-tube rack, an additional sample was included for quality control [17,18].

Bruker DRX600 spectrometer (Bruker Biospin) operating at 600 MHz was used for acquiring all ^1^H NMR spectra. Standard water-suppressed 1D spectrum (Nuclear Overhauser Effect Spectroscopy [NOESY]) and a Carr-Purcell-Meiboom-Gill spectrum were obtained for each sample [17].

The spectra were automatically phased and baseline corrected, and the chemical shifts were calibrated to the glucose signal at 5.233 ppm using TOPSPIN 3.1 (Bruker Biospin). Spectral data were imported into MATLAB version 8.3 (R2014a) (Mathworks Inc.), spectral intensities organized into rectangular matrices (samples as rows, chemical shifts as columns), the regions containing the residual water peak (4.5–5.0 ppm) and those containing only baseline were removed (the spectral range from 0.5 to 10 ppm was kept). Further processing was performed, including peak alignment and normalization using the recursive segment-wide peak alignment [19] and probabilistic quotient normalization [20] methods, respectively.

Metabolites were annotated using with the aid of additional spectral information gathered from 2D NMR experiments [2D J-resolved (2D JRES), COSY (Correlation SpectroscopY), TOCSY (TOtal Correlation SpectroscopY), HSQC (Heteronuclear single quantum correlation spectroscopy)] and statistical correlation methods [STOCSY (Statistical Total Correlation Spectroscopy) and STORM (Subset Optimisation by Reference Matching)]. This information was then compared with available in-house and publicly available databases (Human Metabolome Database) and published data on human serum and plasma metabolite components. Spike-in experiments were used to confirm metabolite identities when feasible. The annotation information was organized into manually defined bins. For this purpose, spectra were divided into smaller spectral regions enclosing each of the detected peaks that were annotated to 1 or more metabolites or macromolecules according to the information gathered using the 2D NMR experiments and statistical correlation methods. Overall, ∼75% of molecules have been annotated with ≥1 associated metabolite.

##### Fasting glucose and insulin

Serum collected at the baseline examination after a minimum 8-h fast was used to measure fasting insulin and glucose. Glucose was measured using the Vitros 950 analyzer (Johnson & Johnson Ortho-Clinical Diagnostics), and insulin was measured using the Linco Human Insulin Specific Radioimmunoassay kit (Linco Research, Inc.).

##### Dysglycemia status

Dysglycemia status was based on American Diabetes Association (ADA) 2003 criteria [21]. T2D was defined as fasting glucose ≥126 mg/dL, or the participant self-reported a previous diagnosis of T2D or medication usage for T2D. For some analyses, those with T2D were stratified into those who had “treated T2D,” i.e., who reported taking any medication for diabetes (including insulin), and “untreated T2D,” which included all those who met the criteria for T2D (known and unknown) who did not report diabetes medication usage. Impaired fasting glucose was defined as a fasting glucose range of 100–125 mg/dL, and normoglycemia was defined as fasting glucose *<*100 mg/dL.

##### Demographics

Age, sex*,* household income, and smoking status were obtained through in-person interviews with trained assessors.

##### Physical activity

Physical activity was assessed using a detailed, semi-quantitative questionnaire adapted from the Cross-Cultural Activity Participation Study (B. Ainsworth, personal communication, San Diego State University).

##### Anthropometric measures

Height and weight were measured by trained study staff. BMI was calculated as weight in kilograms (kg) divided by height in meters (m) squared (kg/m^2^).

### Analyses

All analyses were conducted using R software version 4.0.5 [22].

#### Participant characteristics

Demographic, dietary, and health information were each calculated as a means ± SD for continuous variables, or total number (unweighted *N*) and percentage (%) for categorical or ordinal variables, stratified by avocado intake status (consumer compared with nonconsumer). Differences in these factors by avocado intake status (consumer compared with nonconsumer) were examined using t-tests for continuous variables and χ^2^ tests of difference for categorical variables, with continuous variables that departed from normality (defined as skew and sample kurtosis values between –1 and +1) transformed to approximate normality using an inverse normal transformation prior to analysis.

#### Metabolome-wide association study

The associations of all spectral features with avocado intake were run using linear regression models specifying each spectral feature as the outcome in separate models, with avocado intake (consumer compared with nonconsumer) as the predictor, and age, race, sex, and data collection site as covariates.

Significance was set at the metabolome-wide significance level (MWSL), defined as the threshold needed to maintain the family-wise error rate at 5%, and ascertained by permutation. Following the procedures described in [23], avocado intake status (consumer compared with nonconsumer) was randomly shuffled among the participants (to simulate the null hypothesis) and used as the outcome in a full metabolome-wide association study (MWAS) for each of 10,000 permutations. The MWSL (α′) was chosen as the highest *P* value that satisfied

$$\alpha =\mathrm{Pr}\left(\min\left\{p\right\}<\;\alpha^{\prime }\right)$$

where α was set at α = 0.05, p denotes the *P* value from the i-th variable, and min{p} denotes the minimum *P* value across all associations across all permutations. Based on this procedure, after 10,000 permutations, the MWSL was set at *P* < 5.3∗10^–7^ (95% CI: 6.23∗10^–7^ – 4.49∗10^–7^).

##### Sensitivity analyses

Any significant associations were reanalyzed with the inclusion of smoking status, income level, education level, total EI, and physical activity level as covariates (fully-adjusted model), and subsequently also with the addition of BMI (fully-adjusted + BMI model).

##### Creation of a metabolomic biomarker of avocado intake

One group of highly-correlated spectral features was associated with avocado intake. As these features shared a single annotation (CH2-lysyl), it is likely they represent the same metabolite, and so a mean across all 3 spectral features to represent the average value of this metabolite was taken. This was used as our metabolite biomarker of avocado intake in subsequent analyses.

##### Specificity of the metabolomic biomarker of avocado intake

To examine the extent that the metabolomic biomarker was specific to avocado intake, and in particular, whether other dietary food groups contributed to its concentration, we ran 48 additional linear regression models, in which each of the other 47 food groups available in MESA (see Supplementary Table 1 for food group descriptions), and then overall dietary quality (as indexed by adherence to a Mediterranean-style diet), were added as predictors to the fully-adjusted model, and subsequently the fully-adjusted + BMI model.

##### Associations with fasting glucose, fasting insulin, and incident T2D

Cross-sectional associations between fasting glucose and insulin for avocado intake status category (consumer compared with nonconsumer), and avocado-related metabolites, were conducted via linear regression models, first in the whole population and subsequently in population subgroups defined by glycemia status. Longitudinal associations with incident T2D for avocado intake status; and avocado-related metabolites were conducted using Cox-proportional hazard models. Person years were accumulated from the date of the baseline clinical examination until the date of the examination at which incident T2D was identified, loss to follow-up occurred, or until the date of the most recent examination. In all analyses, adjusted HRs were used to quantify the strength of any significant associations.

##### Sensitivity analyses

In both the linear regression models and Cox-proportional hazard models, 3 sets of models were run: minimally adjusted, fully-adjusted, and fully-adjusted + BMI models, using the same covariates as above.

##### Analyses stratified by dysglycemia status

All associations were additionally examined in subgroups of the overall population, defined by glycemia status (normoglycemia compared with impaired fasting glucose compared with T2D).

## Results

### Participant characteristics

Our sample was comprised of 6220 adults (*N* = 3438 with metabolomic data), of whom 35% (*N* = 2182) reported consuming avocado. Avocado consumers consumed a mean of 0.81 (±1.27) servings of avocado per week. Avocado consumers were younger (t = 8.7, df = 4416, *P* < 2.0 ∗ 10^–16^; Table 1), had completed a higher level of education (χ^2^ = 141, df = 8, *P* < 2.0 ∗ 10^–16^; Table 1) and had a different race/ethnicity distribution (greater proportion of Hispanic individuals) (χ^2^ = 1027, df = 3, *P* < 2.0 ∗ 10^–16^; Table 1) than nonconsumers. Avocado consumers also reported a higher daily EI (t = –7.9, df = 4416, *P* = 4.0 ∗ 10^–16^; Table 1) and higher alcohol consumption (t = –3.8, df = 4177, *P* = 0.0001; Table 1). Consumers and nonconsumers did not differ in terms of sex distribution, income level, smoking status, physical activity, or BMI (all *P* > 0.05; Table 1).

**TABLE 1:** Demographic, anthropometric, and clinical characteristics of participants

	Avocado intake status	
	Nonconsumer (*N* = 4038)	Consumer (*N* = 2182)
Sociodemographic factors		
Age (y)∗∗∗	63.1 (10.15)	60.74 (10.29)
Sex		
Female	2111 (52.3%)	1176 (53.9%)
Male	1927 (47.8%)	1006 (46.1%)
Race/ethnicity (%)∗∗∗		
White	1671 (41.4%)	795 (36.4%)
Chinese	635 (15.7%)	156 (7.1%)
Black	1322 (32.7%)	282 (12.9%)
Hispanic	410 (10.2%)	949 (43.5%)
Educational level (%)∗		
No schooling	32 (0.8%)	36 (1.7%)
Grades 1–8	301 (7.5%)	338 (15.5%)
Grades 9–11	286 (7.1%)	128 (5.9%)
Completed high school/GED	795 (19.7%)	316 (14.5%)
Some college, but no degree	662 (16.4%)	330 (15.2%)
Technical school certificate	301 (7.5%)	130 (6.0%)
Associate degree	204 (5.1%)	119 (5.5%)
Bachelor’s degree	741(18.4%)	344 (15.8%)
Graduate or professional school	708 (17.6%)	441 (20.2%)
Income level (%)		
<$25,000	1200 (31.0%)	666 (31.2%)
$25,000–$49,000	1129 (29.2%)	600 (28.1%)
≥$50,000	1537 (39.8%)	867 (40.7%)
*Health behaviors*		
Smoking status (%)		
Never	2042 (50.2%)	1132 (51.9%)
Former	1491 (37.0%)	782 (35.8%)
Current	499 (12.4%)	268 (12.3%)
EI (kcals/d)∗	1579.0 (813.4)	1761.0 (895.9)
Alcohol consumption (g/d)∗∗∗	4.80 (12.5)	6.14 (13.52)
Diet quality, Mediterranean-style diet score∗∗∗	4.47 (1.92)	4.65 (1.95)
Physical activity (MET-min/wk)	5585 (6037)	5712 (5585)
*Clinical characteristics*		
BMI (kg/m^2^)	28.21 (5.48)	28.23(5.24)
Dysglycemia	97.46 (28.63)	99.43 (35.43)
Normoglycemia	2949 (73.1%)	1,641 (75.3%)
Impaired fasting glucose	575 (14.25%)	280 (12.8%)
Untreated T2D	110 (2.7%)	49 (2.2%)
Treated T2D	400 (9.9%)	210 (9.63)

MET-min: metabolic unit minute equivalents; T2D, type 2 diabetes; GED. General Educational Development.

∗*P* < 0.05, ∗∗*P* < 0.01, ∗∗∗*P* < 0.001 in tests of difference between avocado consumers and nonconsumers.

#### MWAS

**FIGURE 2:**
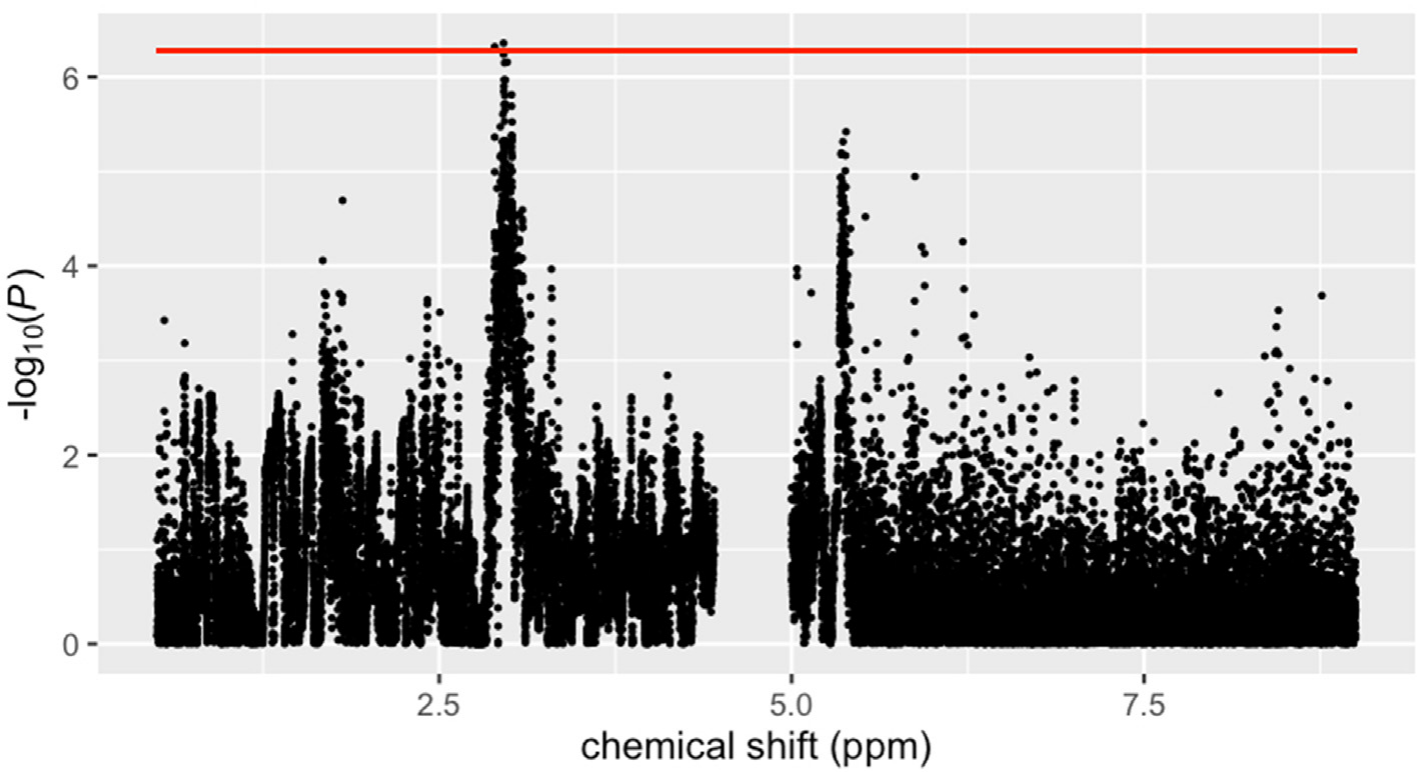
Manhattan plot for associations between avocado intake status (consumer compared with nonconsumers) and Spectral features from the metabolome-wide association study. Line denotes threshold for metabolome-wide significance level (*P* < 5.33 ∗ 10^–7^), determined via permutation analysis (*N* = 10,000 permutations). Analyses controlled for age, race, sex, daily EI, and data collection site as fixed effects.

Across the whole population, 3 spectral features were associated with avocado intake in the full MWAS at metabolome-wide significance levels (all *P* < 5.3 ∗ 10^–7^; Supplemental Table 2; Figure 2), all annotated as CH2-lysyl.

#### Sensitivity analyses

The associations were only slightly attenuated in the fully-adjusted model (e.g., *β* = 0.17 ± 0.04, *P* = 1.1 ∗ 10^–5^ for the feature with the strongest association, as indicated by the lowest *P* value, i.e., the sentinel hit; Table 2) and the fully-adjusted + BMI model (*β* = 0.15 ± 0.04, *P* = 7.7 ∗ 10^–5^ for the sentinel hit; Table 2).

**TABLE 2:** Standardized parameter estimates for associations between avocado intake status (consumer compared with nonconsumer) and spectral features for molecules reaching metabolome-wide significance levels for the whole Multi-Ethnic Study of Atherosclerosis cohort and stratified by dysglycemia status

ppm	Overall population (*N* = 3438)		Normoglycemia (*N* = 2478)		Impaired fasting Glucose (*N* = 521)		Type 2 diabetes			
							Untreated (*N* = 92)		Treated (*N* = 344)	
	*β* (SE)	*P* value	*β* (SE)	*P* value	*β* (SE)	*P* value	*β* (SE)	*P* value	*β* (SE)	*P* value
Metabolome-wide association study^1^										
2.893784194	0.20 (0.04)	1.8 ∗ 10^–7^	0.19 (0.04)	1.4 ∗ 10^–5^	0.29 (0.10)	0.004	0.15 (0.23)	0.53	0.06 (0.13)	0.63
2.956360656	0.20 (0.04)	4.4 ∗ 10^–7^	0.19 (0.05)	3.6 ∗ 10^–5^	0.29 (0.10)	0.005	0.15 (0.23)	0.50	0.03 (0.13)	0.82
2.956697088	0.20 (0.04)	5.2 ∗ 10^–7^	0.20 (0.05)	2.5 ∗ 10^–5^	0.27 (0.10)	0.01	0.19 (0.24)	0.42	0.02 (0.13)	0.90
Fully-adjusted model^2^										
2.893784194	0.17 (0.04)	1.1 ∗ 10^–5^	0.17 (0.05)	1.0 ∗ 10^–4^	0.29 (0.10)	0.005	–0.08 (0.27)	0.78	0.02 (0.13)	0.88
2.956360656	0.17 (0.04)	3.2 ∗ 10^–5^	0.17 (0.05)	4.6 ∗ 10^–4^	0.29 (0.11)	0.01	–0.03 (0.24)	0.91	–0.01 (0.13)	0.96
2.956697088	0.17 (0.04)	2.6 ∗ 10^–5^	0.17 (0.04)	2.8 ∗ 10^–4^	0.27 (0.11)	0.01	–0.04 (0.25)	0.88	–0.03 (0.13)	0.84
Fully-adjusted model + BMI model^3^										
2.893784194	0.15 (0.04)	7.7 ∗ 10^–5^	0.18 (0.05)	1.0 ∗ 10^–4^	0.29 (0.10)	0.005	–0.07 (0.27)	0.80	0.03 (0.12)	0.80
2.956360656	0.14 (0.04)	2.3 ∗ 10^–4^	0.13 (0.05)	0.004	0.29 (0.11)	0.01	–0.01 (0.24)	0.97	0.002 (0.13)	0.98
2.956697088	0.14 (0.04)	1.9 ∗ 10^–4^	0.14 (0.05)	0.002	0.27 (0.11)	0.01	–0.01 (0.25)	0.95	–0.02 (0.13)	0.90

1 Minimally adjusted models controlled for age, sex, race/ethnicity, and data collection site.

2 Fully adjusted models controlled for age, sex, race/ethnicity, data collection site, total EI, education level, income, alcohol intake (g/d), physical activity, and smoking status (current/former/never).

3 Fully adjusted + BMI models controlled for age, sex, race/ethnicity, data collection site, total EI, education level, income, alcohol intake (g/d), physical activity, smoking status (current/former/never), and BMI.

#### Creation of a metabolomic biomarker of avocado intake

All 3 spectral features associated with avocado intake were highly-correlated (Pearson correlations *r* = 0.77–0.97; all *P* < 0.001).

#### Specificity of the metabolomic biomarker of avocado intake

A mean score across all 3 features was significantly associated with avocado intake in the fully-adjusted model (*β* = 0.18 ± 0.04, *P* = 7.9 ∗ 10^–6^). When BMI was added to the model (the fully-adjusted + BMI model; Supplemental Table 3), BMI was strongly associated with the avocado biomarker (*β* = 0.29 ± 0.02, *P* < 2.0 ∗ 10^–16^; Supplemental Table 3), but the association with avocado intake was not markedly attenuated (*β* = 0.15 ± 0.04, *P* = 6.1 ∗ 10^–5^; Supplemental Table 3). There was no evidence that the metabolomic biomarker was associated with physical activity, EI, alcohol intake, or smoking status (all *P* > 0.05; Supplemental Table 3). In models which individually added other forms of dietary intake the models, the metabolomic biomarker was not associated with any of the 47 other food groups nor with dietary quality (all *P* > 0.01; Supplementary Table 4).

#### Associations with fasting glucose, fasting insulin, and incident T2D

Avocado intake was inversely associated with fasting insulin in analyses of the whole population in the fully-adjusted model (*β* = –0.07 ± 0.03, *P* = 0.03; Table 3), but this association was not significant in the fully-adjusted + BMI model (*β* = –0.02 ± 0.03, *P* = 0.37; Table 3). There were no associations between avocado intake and fasting glucose. Over the assessment period for the current analyses, there were 1600 cases of incident T2D. Avocado intake was not associated with incident T2D in any analyses (all *P* > 0.05; Table 3).

**TABLE 3:** Standardized parameter estimates for cross-sectional associations between baseline avocado intake category (consumer compared with nonconsumer) with fasting glucose and insulin, as well longitudinal associations with incident type 2 diabetes, for the whole Multi-Ethnic Study of Atherosclerosis cohort, and stratified by dysglycemia status

Model	Overall population (*N* = 6220)		Normoglycemia (*N* = 4590)		Impaired fasting glucose (*N* = 855)		Type 2 diabetes			
							Untreated (*N* = 159)		Treated (*N* = 610)	
	*β* (SE)	*P* value	*β* (SE)	*P* value	*β* (SE)	*P* value	*β* (SE)	*P* value	*β* (SE)	*P* value
**Cross-sectional association models**										
Fasting glucose										
Minimally adjusted model^1^	–0.07 (0.03)	0.01	–0.03 (0.03)	0.45	0.03 (0.09)	0.70	0.20 (0.19)	0.29	–0.03 (0.10) (0.13)	0.48
Fully-adjusted model^2^	–0.03 (0.03)	0.24	–0.004 (0.03)	0.91	0.09 (0.09)	0.29	0.26 (0.21)	0.22	–0.07 (0.13)	0.82
Fully-adjusted + BMI^3^	–0.01 (0.03)	0.68	0.01 (0.03)	0.66	0.11 (0.09)	0.21	0.25 (0.21)	0.22	–0.08 (0.10)	0.46
Fasting insulin										
Minimally adjusted model^1^	–0.05 (0.03)	0.05	–0.03 (0.03)	0.28	–0.07 (0.08)	0.38	–0.25 (0.19)	0.25	0.07 (0.10)	0.50
Fully-adjusted model^2^	–0.07 (0.03)	0.03	–0.05 (0.04)	0.15	–0.08 (0.08)	0.35	–0.33 (0.21)	0.11	0.03 (0.10)	0.79
Fully-adjusted + BMI^3^	–0.02 (0.03)	0.37	–0.01 (0.03)	0.84	–0.03 (0.07)	0.72	–0.31 (0.18)	0.09	0.01 (0.09)	0.93

**Cox-proportionate hazard models**										
	HR (95% CIs)	*P* value	HR (95% CIs)	*P* value	HR (95% CIs)	*P* value	HR (95% CIs)	*P* value	HR (95% CIs)	*P* value
Incident type 2 diabetes										
Minimally adjusted model^1^	0.91 (0.81–0.10)	0.13	1.00 (0.80–0.16)	0.97	1.11 (0.86–1.44)	0.13	-	-	-	-
Fully-adjusted model^2^	0.94 (0.93–1.07)	0.36	1.03 (0.81–1.32)	0.78	1.21 (0.92–1.59)	0.17	-	-	-	-
Fully-adjusted + BMI model^3^	1.00 (0.89–1.13)	0.95	1.12 (0.89–1.42)	0.33	1.26 (0.95–1.66)	0.10	-	-	-	-

1 Minimally adjusted models controlled for age, sex, race/ethnicity, and data collection site.

2 Fully adjusted models controlled for age, sex, race/ethnicity, data collection site, total EI, education level, income, alcohol intake (g/d), physical activity, and smoking status (current/former/never).

3 Fully adjusted + BMI models controlled for age, sex, race/ethnicity, data collection site, total EI, education level, income, alcohol intake (g/d), physical activity, smoking status (current/former/never), and BMI.

In the fully-adjusted model, the biomarker of avocado intake was significantly associated with lower fasting glucose (*β* = –0.28 ± 0.02, *P* < 2.0 ∗ 10^–16^; Table 4), lower fasting insulin (*β* = –0.31 ± 0.02, *P* < 2.0 ∗ 10^–16^; Table 4), and a lower rate of incident T2D (HR: 0.62; 95% CI: 0.57–0.67; *P* < 2.0 ∗ 10^–16^; Table 4). There was little attenuation of the associations when additionally controlling for BMI (fasting glucose: *β* = –0.22 ± 0.02, *P* < 2.0 ∗ 10^–16^; fasting insulin: *β* = –0.17 ± 0.02, *P* < 2.0 ∗ 10^–16^, rate of incident T2D: HR: 0.68; 0.63–0.64; *P* < 2.0 ∗ 10^–16^; Table 4).

**TABLE 4:** Standardized parameter estimates for cross-sectional associations between a biomarker of avocado intake (mean of 3 spectral features) with average glucose concentrations, fasting glucose, and insulin, as well as longitudinal Associations with incident T2D, for the whole Multi-Ethnic Study of Atherosclerosis population and stratified by dysglycemia status

Model	Overall population (*N* = 3438)		Normoglycemia (*N* = 2478)		Impaired fasting Glucose (*N* = 521)		Type 2 diabetes			
							Untreated (*N* = 92)		Treated (*N* = 344)	
	*β* (SE)	*P* value	*β* (SE)	*P* value	*β* (SE)	*P* value	*β* (SE)	*P* value	*β* (SE)	*P* value
**Cross-sectional association models**										
Fasting glucose										
Minimally adjusted model^1^	–0.29 (0.02)	<2.0 ∗ 10^–16^	–0.19 (0.02)	<2.0 ∗ 10^–16^	–0.07 (0.05)	0.14	–0.13 (0.13)	0.30	–0.23 (0.13)	0.62
Fully-adjusted model^2^	–0.28 (0.02)	<2.0 ∗ 10^–16^	–0.18 (0.02)	<2.0 ∗ 10^–16^	–0.07 (0.05)	0.13	–0.18 (0.16)	0.26	–0.23 (0.06)	9.0 ∗ 10^–5^
Fully-adjusted + BMI model^3^	–0.22 (0.02)	<2.0 ∗ 10^–16^	–0.13 (0.02)	1.5 ∗ 10^–10^	–0.06 (0.05)	0.28	–0.24 (0.16)	0.15	–0.22 (0.06)	3.5 ∗ 10^–4^
Fasting insulin										
Minimally adjusted model^1^	–0.31 (0.02)	<2.0 ∗ 10^–16^	–0.29 (0.02)	<2.0 ∗ 10^–16^	–0.15 (0.04)	8.0 ∗ 10^–4^	–0.17 (0.13)	0.19	–0.28 (0.05)	3.3 ∗ 10^–7^
Fully-adjusted model^2^	–0.31 (0.02)	<2.0 ∗ 10^–16^	–0.29 (0.02)	<2.0 ∗ 10^–16^	–0.13 (0.04)	0.003	–0.24 (0.16)	0.15	–0.30 (0.06)	2.6 ∗ 10^–7^
Fully-adjusted + BMI model^3^	–0.17 (0.02)	<2.0 ∗ 10^–16^	–0.17 (0.02)	<2.0 ∗ 10^–16^	–0.07 (0.04)	0.10	–0.14 (0.15)	0.36	–0.22 (0.05)	5.8 ∗ 10^–5^

**Cox-proportionate hazard models**										
	HR (95% CIs)	*P* value	HR (95% CIs)	*P* value	HR (95% CIs)	*P* value	HR (95% CIs)	*P* value	HR (95% CIs)	*P* value
Incident type 2 diabetes										
Minimally adjusted^1^	0.61 (0.57–0.66)	<2.0 ∗ 10^–16^	0.69 (0.60–0.80)	<2.4 ∗ 10^–7^	0.80 (0.69–0.93)	0.003	-	-	-	-
Fully adjusted^2^	0.62 (0.57–0.67)	<2.0 ∗ 10^–16^	0.68 (0.59–0.79)	<2.3 ∗ 10^–7^	0.82 (0.64–0.89)	0.001	-	-	-	-
Fully-adjusted + BMI^3^	0.68 (0.63–0.74)	<2.0 ∗ 10^–16^	0.80 (0.68–0.93)	0.004	0.79 (0.66–0.94)	0.005	-	-	-	-

1 Miminally adjusted models controlled for age, sex, race/ethnicity, and data collection site.

2 Fully adjusted models controlled for age, sex, race/ethnicity, data collection site, total EI, education level, income, alcohol intake (g/d), physical activity, and smoking status (current/former/never).

3 Fully adjusted + BMI models controlled for age, sex, race/ethnicity, data collection site, total EI, education level, income, alcohol intake (g/d), physical activity, smoking status (current/former/never), and BMI.

#### Analyses stratified by dysglycemia status

The significant associations between avocado intake and spectral features were observed in the participants with normoglycemia in both the fully-adjusted model (*β* = 0.13 ± 0.05, *P* = 0.004 for the sentinel hit; Table 2) and the fully adjusted + BMI model (*β* = 0.14 ± 0.05, *P* = 0.002 for the sentinel hit; Table 2), and in those with impaired fasting glucose in both the fully-adjusted model (*β* = 0.29 ± 0.11, *P* = 0.01 for the sentinel hit; Table 2), and the fully-adjusted + BMI model (*β* = 0.27 ± 0.11, *P* = 0.01 for the sentinel hit; Table 2). No associations were observed in those with T2D (all *P* > 0.05; Table 2).

Associations between avocado intake and incident T2D of a similar magnitude but which did not reach significance were seen in the subgroup analyses (all *P* > 0.05), except for those with treated T2D (Table 3).

The glycemia subgroup analyses suggested that the association between avocado and the 3 avocado-related features observed in the population as a whole were driven by those with normoglycemia (e.g., *β* = 0.17 ± 0.04, *P* = 2.8 ∗ 10^–4^ for the sentinel feature in the fully-adjusted model; Table 2) and those with impaired fasting glucose (e.g., *β* = 0.27 ± 0.11, *P* = 0.01 for the sentinel feature in the fully-adjusted model; Table 2). We did not find evidence of an association between the sentinel features of interest and avocado intake in those with T2D, whether the T2D was untreated (e.g., *β* = –0.08 ± 0.27, *P* = 0.78) for the sentinel feature in the fully-adjusted model; Table 2) or treated (e.g., *β* = 0.02 ± 0.13, *P* = 0.88).

#### Biomarker and glycemia

Similar directions and magnitudes of effects were observed in the dysglycemia subgroups, although these did not reach significance in the small number (*N* = 92) of participants with untreated T2D (Table 4).

## Discussion

Using data from MESA, a population-based cohort of United States adults with diverse ancestry, the current study investigated associations between avocado-related metabolites and glycemia, including incident T2D. In our analyses, there was a modest association between self-reported avocado intake and fasting insulin that was not significant when controlling for BMI. However, metabolomic markers of avocado intake, identified via our MWAS, showed strong and highly significant associations with lower fasting glucose and insulin and lower rates of incident T2D. These associations persisted when controlling for sociodemographic factors, health behaviors, and also for adiposity (i.e., BMI).

Several components of avocado are thought to support glucose homeostasis. For example, supplementation with avocatin B, a bioactive compound derived from avocado lipid, improved glucose tolerance and glucose utilization and reduced insulin resistance in mice with diet-induced obesity [24]. Avocado is also high in MUFA and relatively high in dietary fiber. MUFA is thought to have a beneficial effect of dietary on insulin sensitivity via conservation of the IRS-1/PI3 kinase insulin signaling pathway [25], whereas a biproduct of fiber digestion is short-chain fatty acids (SCFAs), such as butyrate [26,27]. Butyrate inhibits histone deacetylases [28,29]; metformin is another histone deacetylase inhibitor [30] with known effects on β-cell differentiation, proliferation, and function, which all contribute to metformin’s effects on reducing insulin resistance [31]. In our previous study, we observed associations between avocado intake and lower fasting insulin, lower HbA1c, a higher insulin sensitivity, as well as lower rates of T2D in a large population of Hispanic/Latino adults [3,4]; findings which converge with those from smaller-scale intervention studies [5,6]. We observed a similar pattern of results in the current study, observing associations with lower fasting insulin that were attenuated when correcting for BMI, but although the observed associations with lower rates of incident T2D were in the same direction, they did not reach significance in the current study.

Previous avocado-glycemia associations have differed between individuals with normoglycemia and dysglycemia. In data from over 16,000 individuals (including ∼1500 with T2D), much of the population-level associations between avocado and glycemia were driven by the participants with untreated T2D: in those with normoglycemia, avocado intake was only modestly associated with a higher insulinogenic index, whereas in those with T2D, avocado intake was associated with lower HbA1c values, lower fasting glucose, better *β* -cell functioning (higher HOMA- %*β* values), higher insulin concentrations 2-h after an oral glucose challenge, and a higher insulinogenic index – associations which were attenuated, but mostly remained significant when controlling for BMI (Senn et al. [3] submitted data). Similarly, in a separate analysis, the association of avocado intake with lower incident T2D was stronger in those with dysglycemia at baseline [4]. Although our power was reduced for subgroup analyses, parameter estimates suggested that our cohort-level associations differ between those with and those without dysglycemia. These prior findings represent an emerging pattern of results across multiple studies, whereby the association of MUFA and MUFA-containing foods with glycemia differ by dysglycemia status [[32], [33], [34], [35]], highlighting the importance of individual differences in diet-health associations.

In the current study, we aimed to better address individual differences in diet-health associations in our estimated avocado-glycemia associations by incorporating biomarkers of avocado, which arise after the digestion, metabolism, and processing of intake – a heterogenous process across individuals. In a metabolome-wide analysis, we identified 3 highly-correlated spectral features associated with avocado intake, which we combined to form a biomarker of avocado intake in our population. These features were not associated with other lifestyle behaviors, such as alcohol intake or physical activity, nor with overall dietary quality or the intake of any of 47 other food groups to the same extent, including the food group “nuts and seeds” (Supplemental Table 4), which contains foods high in MUFA (Supplemental Table 1). This suggests the specificity of our metabolomic biomarker to avocado intake within the diet. Further, although future studies are needed to understand the biochemical pathways these features fall along, we observed highly significant associations between a mean score across all 3 features and both lower fasting glucose and lower fasting insulin. Further, the feature score showed a protective association with risk of developing T2D, such that the odds of developing T2D were ∼1.5 times higher for each SD decrease in the mean feature score.

Our finding that our biomarker of avocado intake showed much stronger associations with glycemia and T2D risk than did the reported avocado intake mirrors our previous finding whereby red meat-associated metabolites showed similarly strong associations with markers of inflammation, relative to those between inflammation and red meat intake (Wood et al. [7] submitted data). What gives rise to these patterns of association, whereby health outcomes show associations with diet-related metabolites that are noticeably stronger than those with reported dietary intake, is not immediately clear. Self-reported dietary data are subject to well-known sources of systematic bias, which do not influence assessments of metabolite concentrations [[36], [37], [38]]. In addition, because of the large number of individuals who did not report consuming avocado, avocado intake was dichotomized, reducing measurement precision. This was not the case for our metabolite biomarker, for which the full distribution of values was represented. Taken together, although metabolomic assays (like all assays) have a margin of error, the differences in the magnitude of associations with health for avocado intake compared with the biomarker of avocado intake could reflect increased measurement precision for the latter. However, when measured in plasma, metabolomic features reflect dietary intake after digestion, processing, and absorption. Thus, any heterogeneity in diet-metabolite associations could reflect differences in how food is metabolized. Although we acknowledge our lack of power in participants with untreated (*N* = 92) and treated T2D (*N* = 344), parameter estimates did provide evidence of an association with avocado intake and our metabolomic biomarker of avocado intake in those with T2D, respectively. This would support, but not demonstrate, the notion that the metabolism of avocado differs by dysglycemia status, which could occur via, for example, changes in the microbiome associated with T2D [[39], [40], [41]]. As our metabolomic biomarker has already subsumed such heterogeneity in the digestive process, this could explain why our hypothesis that metabolite-glycemia associations would be stronger in those with dysglycemia was not supported, despite multiple lines of evidence showing different associations between glycemia and MUFA-containing foods (including avocados [3,4]) and glycemia, according to dysglycemia status [[20], [21], [22], [23]].

Our study benefited from the use of untargeted metabolomic data, which allowed for a hypothesis-free approach. However, our ability to annotate all molecules of interest was limited, which remains an important future direction. In addition, our key dietary factor, avocado, is an episodically consumed food [9], and intake for the current analyses was ascertained via self-report. While FFQs are particularly suitable for assessing the intake of episodically consumed foods compared to, for example, 24-h dietary recalls or food diaries, the large number of nonconsumers in our data necessitated dichotomizing intake in avocado “consumers” and “nonconsumers.” This decision improved the robustness of our inferential statistics, but we may have misclassified some consumers as nonconsumers. While such nonconsumers are likely to consume avocado extremely infrequently, and therefore the effects of avocado on the fasting metabolome and on health conditions that develop over the long term, such as T2D, are likely to be negligible, this nonetheless introduced a source of error and over above the expected errors when using self-reported dietary intake data, and could contribute to the small observed associations of glycemia with avocado intake, as compared to our metabolites [[36], [37], [38]]. Finally, as with all observational studies, it was also not possible to ensure that all confounders (including both measured and unmeasured factors) were accounted for, which precludes causal inferences.

In conclusion, in the first study to use metabolomics data to understand links between avocado intake and glycemia, we found that molecules associated with avocado intake may differ by dysglycemia status. However, the strong associations of these molecules with lower fasting glucose and insulin, and a lower incidence of T2D is observed regardless of dysglycemia. In addition to confirming the metabolite annotation, future studies should investigate which factors moderate associations between dietary intake and the metabolome. Nonetheless, our analyses contribute to a growing body of work demonstrtaing that diet-health investigations benefit from metabolomic data, which serve as individualized biomarkers of food intake after digestion, metabolism, and absorption.

## Funding

This work was funded by a grant from The Hass Avocado Board to ACW. Hass Avocado Board did not have input into the study design, analysis, or interpretation of results. ACW and MKS are also supported, in part, by USDA/ARS (Cooperative Agreement 58-3092-5-001). The contents of this publication do not necessarily reflect the views or policies of the USDA, nor does mention of trade names, commercial products, or organizations imply endorsement from the United States government.

The Multi-Ethnic Study of Atherosclerosis (MESA) projects are conducted and supported by the NHLBI in collaboration with MESA investigators. Support for MESA is provided by contracts 75N92020D00001, HHSN268201500003I, N01-HC-95159, 75N92020D00005, N01-HC-95160, 75N92020D00002, N01-HC-95161, 75N92020D00003, N01-HC-95162, 75N92020D00006, N01-HC-95163, 75N92020D00004, N01-HC-95164, 75N92020D00007, N01-HC-95165, N01-HC-95166, N01-HC-95167, N01-HC-95168, N01-HC-95169, UL1-TR-000040, UL1-TR-001079, UL1-TR-001420, UL1TR001881, DK063491, R01HL133932 and R01HL105756. Additional support for the metabolomics data was provided by NIH/NHLBI (R01HL133932) and the EU COMBI-BIO project (FP7, 305422). ACW, MOG, and JIR roles in the current study were funded, in part, by NIH grants from the NIDDK (R01-DK109588, P30-DK063491) and from the National Center for Advancing Translational Sciences (UL1TR001420, UL1TR001881). MOG was also supported by the Eris M. Field Chair in Diabetes Research.

## Author contributions

The authors’ responsibilities were as follows– ACW: designed the research, analyzed the data, wrote the paper, and had final responsibility for the content; MKS, MDG, MAA, and MYM: reviewed the findings and provided novel insights into their interpretation; TE and RPT: conducted the research and provided essential laboratory services; GG, PG, and DMH: conducted the research, provided essential laboratory services, and contributed substantially to writing the manuscript and to the interpretation of results; IT and PE: provided essential laboratory services; MOG and JIR: provided essential expertise, contributed to the interpretation of results, and substantially contributed to writing the manuscript and all authors: read and approved the final manuscript.

## Conflict of interest

ACW has received funding from Sabra Dipping Company, NCBA (The National Cattlemen’s Beef Association), and Ionis Pharmaceuticals for studies unrelated to the current analyses. MOG served on an advisory board for Nestle Health Science. All other authors report no conflicts of interest.

## Data availability

All data, except for the untargeted ^1^H NMR spectral features, are publicly available from the Database of Genotypes and Phenotypes (dbGap; URL: https://www.ncbi.nlm.nih.gov/gap/). The untargeted ^1^H NMR spectral features are available via the MESA data coordinating center, pending the approval of a manuscript proposal and the completion of a data and materials distribution agreement (DMDA; please see https://internal.mesa-nhlbi.org/).

## Appendix A Supplementary data

Multimedia component1 Multimedia component1
